# Effect of intraoperative PEEP with recruitment maneuvers on the occurrence of postoperative pulmonary complications during general anesthesia––protocol for Bayesian analysis of three randomized clinical trials of intraoperative ventilation

**DOI:** 10.12688/f1000research.125861.2

**Published:** 2023-06-23

**Authors:** Guido Mazzinari, Fernando G. Zampieri, Lorenzo Ball, Niklas S. Campos, Thomas Bluth, Sabrine N.T. Hemmes, Carlos Ferrando, Julian Librero, Marina Soro, Paolo Pelosi, Marcelo Gama de Abreu, Marcus J. Schultz, Ary Serpa Neto, PROVHILO investigators, iPROVE investigators, PROBESE investigators, PROVE network investigators

**Affiliations:** 1 Perioperative Medicine, Instituto de Investigación Sanitaria la Fe, Valencia, Spain, 46026, Spain; 2 Anesthesiology, Hospital Universitario y Politécnico la Fe, Valencia, Spain, 46026, Spain; 3 Academic Research Organization, Albert Einstein Hospital, Sao Paulo, Brazil; 4 Surgical sciences and integrated diagnostics, University of Genoa, Genoa, Italy; 5 IRCCS Policlinico San Martino, Genoa, Italy; 6 Critical Care Medicine, Hospital Israelita Albert Einstein, Sao Paulo, Brazil; 7 Cardio pulmonary department, Instituto do Coração, Hospital das Clinicas HCFMUSP, Faculdade de Medicina, Universidad de de Sao Paulo, Sao Paulo, Brazil; 8 Pulmonary Engineergin group, Anesthesiology and intensive Care, University Hospital Carl Gustav Carus, Technische Universität Dresden, Dresden, Germany; 9 Anesthesiology, Amsterdam University Medical Centers, location ‘AMC’, Amsterdam, The Netherlands; 10 Intensive Care, Amsterdam University Medical Centers, location ‘AMC’, Amsterdam, The Netherlands; 11 Anesthesiology and Critical Care, Hospital Clinic de Barcelona, Institut D'investigació August Pi i Sunyer, Barcelona, Spain; 12 CIBER (Center of Biomedical Research in Respiratory Diseases, Instituto de Salud Carlos III, Madrid, Spain; 13 Red de Investigación en Servicios de Salud en Enfermedades Crónicas (REDISSEC), Pamplona, Spain; 14 INCLIVA Clinical Research Institute, Hospital Clinico Universitario de Valencia, Valencia, Spain; 15 Nuffield Department of Medicine, University of Oxford, Oxford, UK; 16 Mahidol Oxford Tropical Medicine Research Unit (MORU), Faculty of tropical medicine, Mahidol University, Bangkok, Thailand; 17 Australian and New Zealand Intensive Care Research Centre (ANZIC-RC), Monash University, Melbourne, Australia; 18 Critical Care, Melbourne Medical School, University of Melbourne, Austin Hospital, Melbourne, Australia

**Keywords:** Mechanical ventilation, intraoperative ventilation, PEEP, recruitment maneuvers, postoperative pulmonary complications, Bayesian analysis

## Abstract

**Background:** Using the frequentist approach, a recent meta–analysis of three randomized clinical trials in patients undergoing intraoperative ventilation during general anesthesia for major surgery failed to show the benefit of ventilation that uses high positive end–expiratory pressure with recruitment maneuvers when compared to ventilation that uses low positive end–expiratory pressure without recruitment maneuvers.

**Methods:** We designed a protocol for a Bayesian analysis using the pooled dataset. The multilevel Bayesian logistic model will use the individual patient data. Prior distributions will be prespecified to represent a varying level of skepticism for the effect estimate. The primary endpoint will be a composite of postoperative pulmonary complications (PPC) within the first seven postoperative days, which reflects the primary endpoint of the original studies. We preset a range of practical equivalence to assess the futility of the intervention with an interval of odds ratio (OR) between 0.9 and 1.1 and assess how much of the 95% of highest density interval (HDI) falls between the region of practical equivalence.

**Ethics and dissemination:** The used data derive from approved studies that were published in recent years. The findings of this current analysis will be reported in a new manuscript, drafted by the writing committee on behalf of the three research groups. All investigators listed in the original trials will serve as collaborative authors.

## Introduction

Mechanical ventilation under general anesthesia and neuromuscular blockade yields a reduction in lung volume that can affect respiratory mechanics and gas exchange, ^1^ especially in specific clinical scenarios such as laparoscopic surgery with pneumoperitoneum insufflation. ^2^ This reduction in volume can lead to a cyclic opening and closing of alveoli during mechanical ventilation, ultimately inducing tissue injury known as atelectrauma. ^3^ Minimizing atelectrauma by applying a ‘lung protective ventilation’ to reopen the closed alveoli with a recruitment maneuver (RM) while sustaining their permeability by applying higher positive end–expiratory pressure (PEEP) is likely associated with a reduction in postoperative pulmonary complications. ^4^ The optimization of intraoperative ventilation and its potential beneficial effects on clinically relevant postoperative outcome measures is of particular importance due to the large number of surgical operations worldwide per year. ^5^

Using the frequentist statistical approach, a recent meta–analysis of three randomized clinical trials (RCTs) in patients undergoing intraoperative ventilation during general anesthesia for major surgery failed to show the benefit of ventilation that uses high PEEP with RM when compared to ventilation that uses low PEEP without RM. ^6^

To enhance comprehension of the study data, the Bayesian methodology is beginning to be used in anesthesiology and critical care studies with uncertain frequentist outcomes. ^7^^–^^10^ In addition, applying a Bayesian framework in meta–analysis allows to model the heterogeneity estimation directly and to estimate pooled effects more precisely, especially when the number of studies included in the analysis is small. ^11^ Furthermore, Bayesian analysis can produce a full posterior distribution for both the effect estimate and heterogeneity and provide the capability of testing for tailored hypotheses assessing, for instance, if the estimate is smaller or larger than a specified interesting threshold. ^12^^,^^13^

We hypothesize that the intervention effect on the posterior probability distribution will lay outside a predefined region of practical equivalence. To test this hypothesis, we will reanalyze the pooled individual patient dataset of the three largest RCTs of intraoperative ventilation comparing ventilation with high PEEP with RM with ventilation with low PEEP without RM using a Bayesian framework according to previously published recommendations. ^7^^,^^14^ The protocol for the Bayesian statistical approach is presented in this paper. The posterior probability for the effect of the intervention on postoperative pulmonary complications will be assessed to better understand the potential benefit or harm of the tested intervention. This could provide a more interpretable probabilistic framework and allows to include preexisting knowledge into the analysis.

## Protocol

### Study type

We will perform a Bayesian analysis of the previously combined dataset named ‘Re–evaluation of the Effects of High PEEP with Recruitment Manoeuvres versus Low PEEP without Recruitment Manoeuvres During General Anaesthesia for Surgery’ (REPEAT), ^6^^,^^15^ registered at ClinicalTrials.gov: NCT03937375 on 3 ^rd^ May 2019. This dataset merged the individual patient data from three RCTs, the ‘High versus low positive end–expiratory pressure during general anesthesia for open abdominal surgery’ (PROVHILO) study (registered with Controlled-Trials.com: ISRCTN70332574), ^16^ the 'Individualized perioperative open–lung approach versus standard protective ventilation in abdominal surgery' (iPROVE) study (registered with ClinicalTrials.gov: NCT02158923) ^17^ and the ‘Effect of intraoperative high positive end–expiratory pressure with recruitment maneuvers vs low PEEP on postoperative pulmonary complications in obese patients’ (PROBESE) study (registered with ClinicalTrials.gov: NCT02148692). ^18^ The study protocols of the original studies were approved by the respective institutional review boards and were published before the start of patient enrolment. ^19^^–^^21^ Written informed consent was obtained from all participating individuals before enrolment, and the rules of good clinical practices were followed. All analyses were performed with R version 4.0.1 (R Core Team).

### Interventions from the original trials

PROVHILO was an international multicenter study comparing intraoperative ventilation with 12 cm H _2_O PEEP with RM to intraoperative ventilation with 0–2 cm H _2_O PEEP without RM in non–obese patients scheduled for major abdominal surgery. ^16^ iPROVE was a national multicenter study comparing two intraoperative ventilation strategies and two postoperative ventilatory support strategies in non-obese patients scheduled for major abdominal surgery. ^17^ In this study, intraoperative ventilation with high PEEP titrated to the best respiratory compliance combined with RM was compared to intraoperative ventilation with 5 cm H _2_O PEEP without RM. Postoperative ventilation was carried out by applying 5 cm H _2_O PEEP continuous positive airway pressure (CPAP) and supplementary oxygen at 0.5 fraction of inspired oxygen (FiO _2_) or 0.5 FiO _2_ alone. PROBESE was an international multicenter study comparing intraoperative ventilation with 12 cm H _2_O PEEP with RM to intraoperative ventilation with 4 cm H _2_O PEEP without RM in obese patients scheduled for major surgery. ^18^

### Data management

The REPEAT database is harmonized, protected, and does not contain any patient–identifying information *.* Data are stored at Hospital Israelita Albert Einstein, Sao Paulo, Brazil. The full description of the data harmonization process is published elsewhere in full detail. ^6^^,^^15^ Briefly, the level of PEEP considered in the low PEEP group was considered as ≤5 cm H _2_O and data from iPROVE were used according to the intraoperative ventilation strategy as no significant interaction with postoperative intervention was found. ^15^

### Outcomes

The single primary outcome of this current analysis is a collapsed composite of postoperative pulmonary complications (PPCs) developed during the first seven postoperative days as collected in the REPEAT database. The definitions used in the study are reported in Table 1.

**Table 1.** Definitions of postoperative pulmonary complications.

	PROVHILO	iPROVE	PROBESE	
Mild respiratory failure	PaO _2_ < 60 mmHg or SpO _2_ < 90% breathing at least 10 minutes of room air but responding to supplemental oxygen of 2 L/minute	SpO _2_ < 92% with FiO _2_ of 0.21 or SpO _2_ < 95% with FiO _2_ of 0.50	PaO _2_ < 60 mmHg or SpO _2_ < 90% breathing at least 10 minutes of room air but responding to supplemental oxygen of 2 L/minute	
Severe respiratory failure	PaO _2_ < 60 mmHg or SpO _2_ < 90% breathing ≥ 10 minutes of room air but responding only to supplemental oxygen > 2 L/minute or need for noninvasive or invasive mechanical ventilation	Increased FiO _2_, increased requirement for CPAP, or the need for noninvasive or invasive ventilation	PaO _2_< 60 mmHg or SpO _2_ < 90% breathing ≥ 10 minutes of room air but responding only to supplemental oxygen > 2 L/minute or need for noninvasive or invasive mechanical ventilation	
ARDS	AECC criteria ^*^	Berlin criteria ^**^	Berlin criteria ^**^	
Pulmonary infection	Need of antibiotics and at least one of the following criteria: new or changed sputum, new or changed lung opacities on chest X-ray when clinically indicated, tympanic temperature >38.3°C, WBC count >12,000/μl in the absence of other infectious focus	Presence of a new pulmonary infiltrate and/or progression of previous pulmonary infiltrates on a chest radiograph plus at least two of the following criteria: (a) leukocytosis with > 12,000 WBC/mm ^3^ or leukopenia with < 4000 WBC/mm ^3^, (b) fever > 38.5°C or hypothermia < 36°C, and (c) increased secretions with purulent sputum and a positive bronchial aspirate	Presence of a new pulmonary infiltrate and/or progression of previous pulmonary infiltrates on a chest radiograph plus at least two of the following criteria: (a) leukocytosis with > 12,000 WBC/mm ^3^ or leukopenia with < 4000 WBC/mm ^3^, (b) fever > 38.5°C or hypothermia < 36°C, and (c) increased secretions with purulent sputum and a positive bronchial aspirate	
Pleural effusion	Chest radiography with the presence of costophrenic angle blunting, displacement of adjacent anatomical structures, and blunting of the hemidiaphragmatic silhouette in the supine position	Chest radiography with the presence of costophrenic angle blunting, displacement of adjacent anatomical structures, and blunting of the hemidiaphragmatic silhouette in the supine position	Chest radiography with the presence of costophrenic angle blunting, displacement of adjacent anatomical structures, and blunting of the hemidiaphragmatic silhouette in the supine position	
Atelectasis	Chest radiography with lung opacification with shift of the mediastinum, hilum, or hemidiaphragm towards the affected area, and compensatory overinflation in the adjacent non-atelectatic lung	Combination of SpO _2_ ≤ 96% during the air test and chest radiography with lung opacification with shift of the mediastinum, hilum, or hemidiaphragm towards the affected area, and compensatory overinflation in the adjacent non-atelectatic lung	Chest radiography with lung opacification with shift of the mediastinum, hilum, or hemidiaphragm towards the affected area, and compensatory overinflation in the adjacent non-atelectatic lung	
Pneumothorax	Chest radiography with air in the pleural space with no vascular bed surrounding the visceral pleura	Chest radiography with air in the pleural space with no vascular bed surrounding the visceral pleura	Chest radiography with air in the pleural space with no vascular bed surrounding the visceral pleura
Bronchospasm	Presence of expiratory wheezing treated with bronchodilator	Presence of expiratory wheezing treated with bronchodilator	Presence of expiratory wheezing

ARDS: Acute Respiratory Distress Syndrome; SIRS: systemic inflammatory response syndrome; AECC: American–European consensus conference; WBC: White blood cells; PaO _2_: Arterial oxygen pressure: SpO _2_: peripheral oxygen saturation; FiO _2_: inspired oxygen fraction.

^*^ Bernard GR, Artigas A, Brigham KL, *et al*. Report of the American-European consensus conference on ARDS: definitions, mechanisms, relevant outcomes and clinical trial coordination. The Consensus Committee. Intensive Care Med 1994;20:225–232.

^**^ Ranieri VM, Rubenfeld GD, Thompson BT, *et al.* Acute respiratory distress syndrome: the Berlin Definition. JAMA 2012;307:2526–2533.

### Power calculation

For this unplanned analysis, we will use all the available data without any *a priori* power calculations.

### Analysis plan

We will carry out a one–stage approach meta–analysis by fitting a multilevel Bayesian logistic model including the effect of the PEEP and RM strategy as population (fixed) effect and the study and site as a varying (random) effect modeling heterogeneity of effect across different studies.

### Prior definition

**Figure 1.**
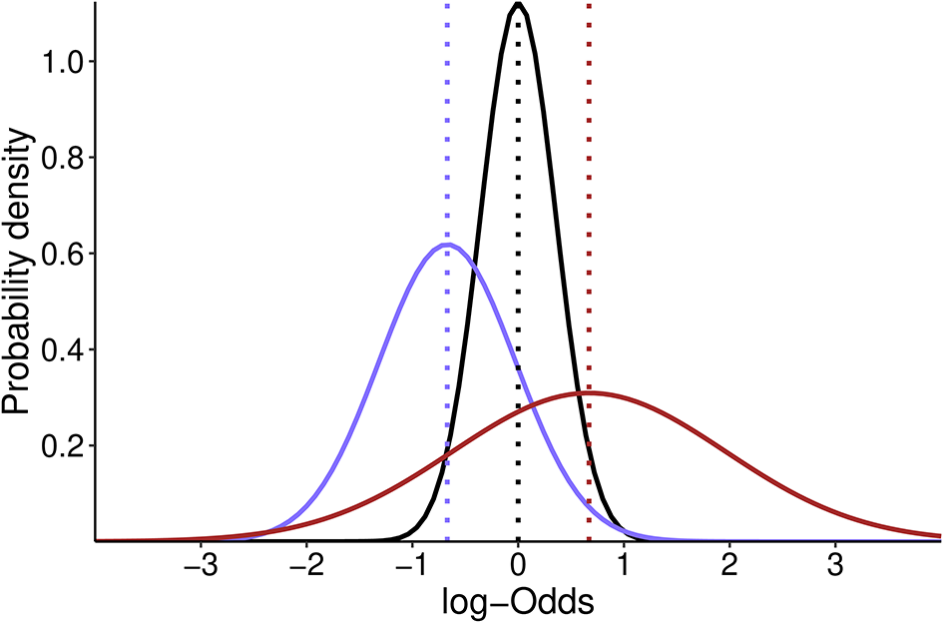
Probability density distribution of the optimistic (purple), skeptic (black) and pessimistic (dark red) prior for the effect estimate in log-Odds scale. Dotted lines show the mean estimate for each distribution. The skeptic prior is centered at 0 while optimistic and pessimistic prior are centered at -0.67 and 0.67 respectively.

**Figure 2.**
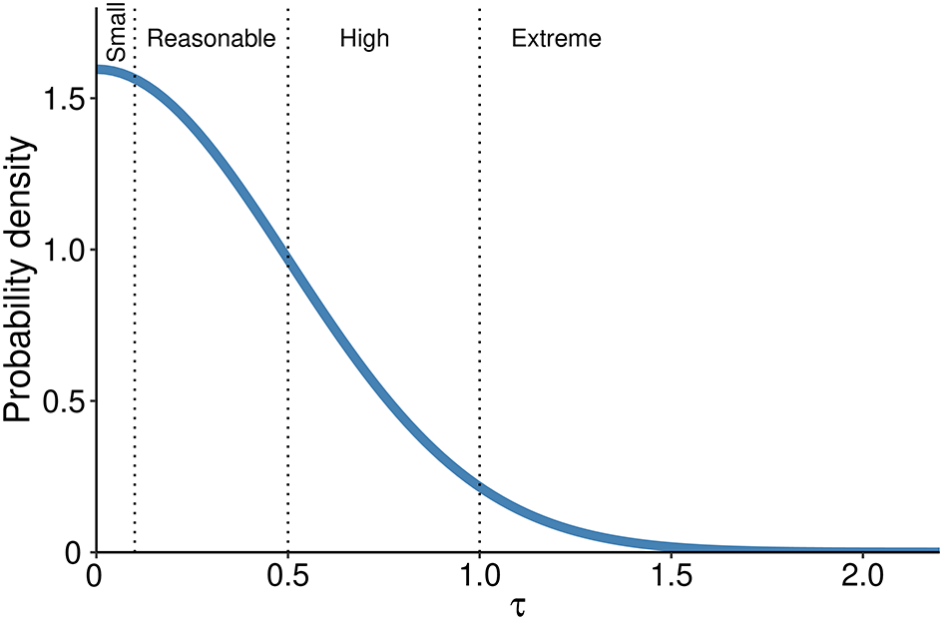
Probability density distribution for the heterogeneity prior. Dotted lines separate heterogeneity categories based on τ values.

**Figure 3.**
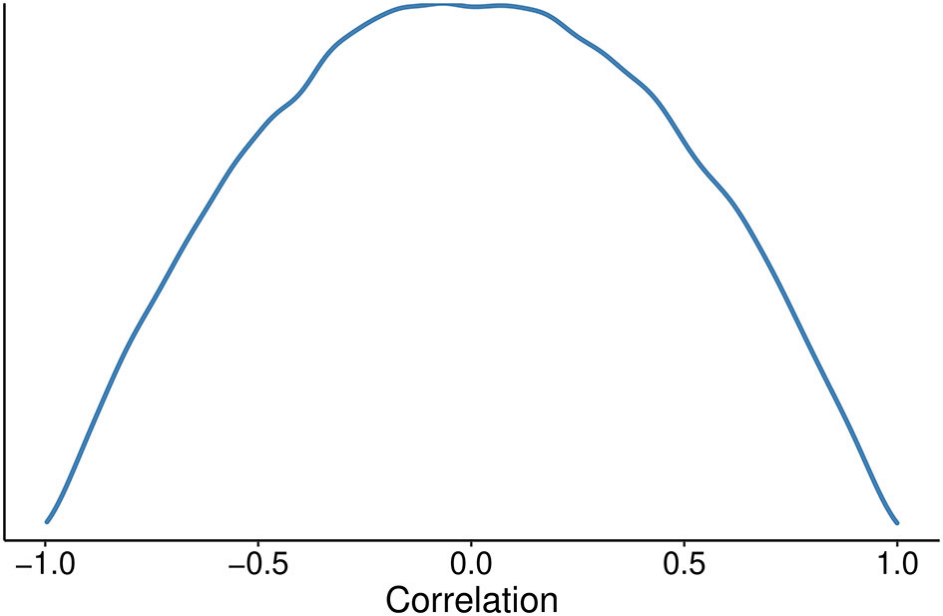
Probability density distribution for the correlation prior.

For defining priors, we will follow previously published recommendations on Bayesian reanalysis ^7^ and recommendations from studies focusing on Bayesian modelling and meta–analysis. ^22^^,^^23^ The guidelines recommend to consider the full range of possible beliefs. Thus, we will use one skeptical, one pessimistic, and one optimistic prior for the effect of PEEP and RM compared to low PEEP, i.e., a PEEP of 5 cm H _2_O or less, without RM on PPCs. In other words, we define three distributions to decide where to assign most of the probability mass. The pessimistic and optimistic prior distributions will be centred, on the averaged estimate from the original, whereas the skeptical prior will be centred around the absence of effect, i.e. 0. The variance of the prior distributions is defined according to the clinical belief that we cannot rule out a benefit for the intervention, although we can likely rule out a large effect and we cannot exclude a probability of harm and we set the probability masses accordingly. Therefore, we will consider a moderate belief strength for both the optimistic and neutral prior and a weak pessimistic prior. Following previous recommendations, ^7^ we will define priors as follows:

• The neutral skeptical prior of moderate strength is normally distributed and centered at the absence of effect [OR = 1; log (OR) = 0] with an SD of 0.355, such that 0.95 of the probability falls in the range of 0.5–2. Therefore, our skeptical prior will follow a normal distribution with a mean of 0 and an SD of 0.355 [N(0, 0.355)] ( Figure 1);
• The pessimistic and optimistic priors are informed by the averaged effect size estimate from the three original studies (PROVHILO: OR = 0.63; iPROVE: OR = 0.45; PROBESE: OR = 0.44; Mean: OR = 0.51). The standard deviation (SD) of the optimistic prior will be defined to retain a 0.15 probability of harm [Pr (OR > 1)], and the pessimistic prior is chosen to retain a 0.30 probability of benefit [Pr (OR < 1)] ( Figure 1);
• The prior for the heterogeneity of the intervention across studies is defined first assuming that at least some between–study variability is present; thus, it should always be more than 0. In the context of log-ORs there are established thresholds of heterogeneity according to the parameter τ that are defined as ‘reasonable’ (0.1 < τ < 0.5), ‘fairly high’ (0.5 < τ < 1.0), ‘fairly extreme’ (τ > 1.0). ^24^ As recommended in a previously published paper ^23^ we assume a prior distribution for heterogeneity as a half-Normal with a mean of 0 and an SD = 0.5, this yields prior probabilities of 52% in the reasonable, 27% in the fairly high and 5% in the extreme category respectively ( Figure 2);
• The prior correlation for the correlation matrix will be based on the Lewandowski-Kurowicka–Joe (LKJ) distribution with a η parameter of 2 for the varying effect. ^22^^,^^25^ ( Figure 3).

### Setting the Range of Practical Equivalence (ROPE)

**Figure 4.**
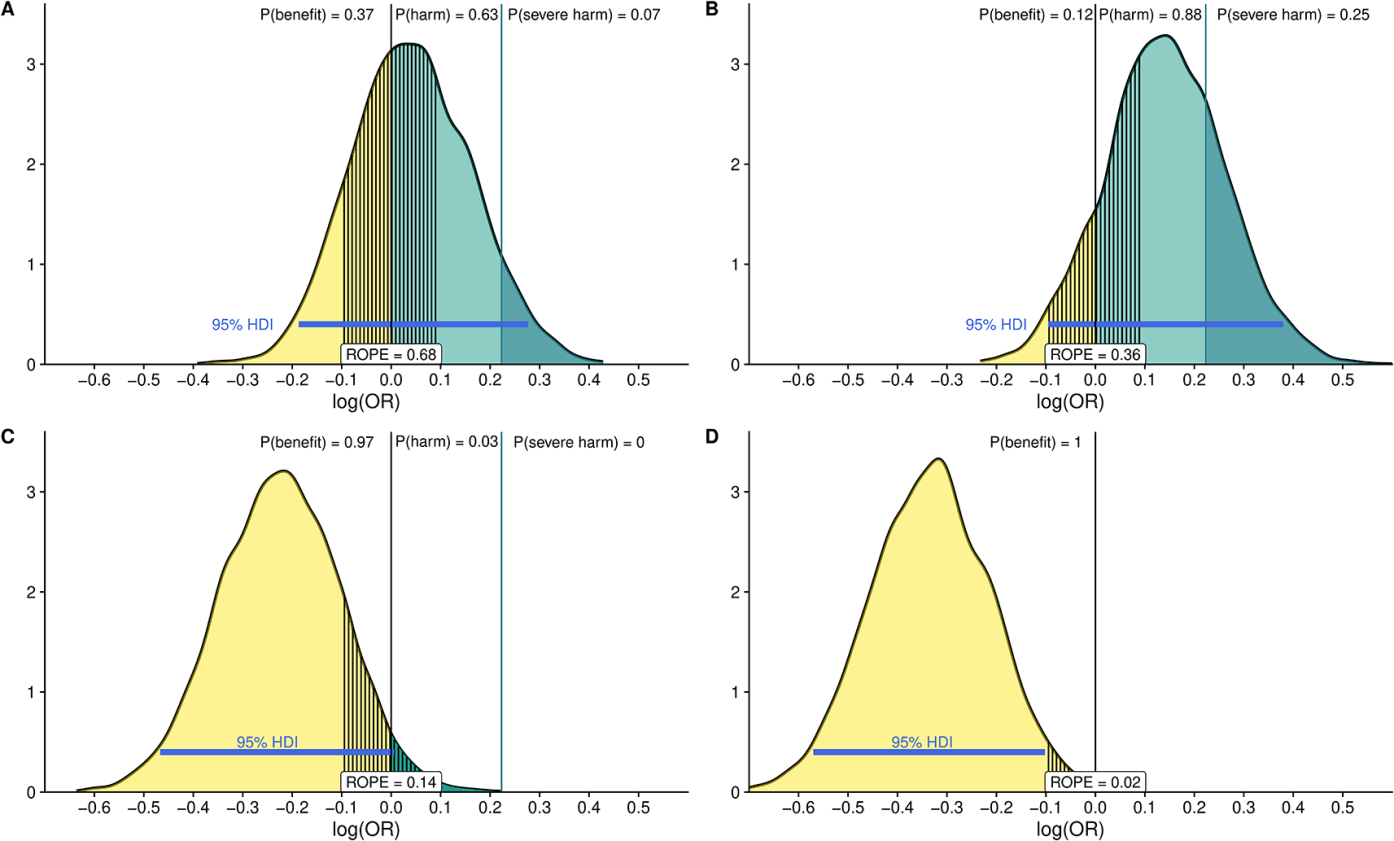
Posterior distribution of the log odds ratio (OR) fitting four logistic models using a “skeptical” prior with differing effect estimates: Panel A: slightly harmful, B: moderately harmful, C: moderately beneficial and D: strongly beneficial The distributions represent 100,000 draws from the posterior. The black vertical line at 0 represents the point at which the OR is equal to 1 [i.e., log (OR) = 0]. The area to the right (in green) represents the probability that the intervention is harmful. The probability of severe harm [Pr (OR.1.25)] is shown in darker green. Probabilities for each posterior distribution are reported in the upper part of each panel. Values below 0 mean the intervention is beneficial [Pr (log(OR),0); Pr (OR,1.0)] and are shown in light yellow with the probability of benefit again in the upper part of the figure. The ROPE is defined as the OR between 1/1.1 and 1.1 (the segmented area around log (OR) = 0). The 95% High-Density Interval (HDI) is reported as a blue line in each panel. We can see how fitting logistic models with different simulated effect yields to different interpretation for instance there is considerable difference between panel A, where the probability of harm is 63% and the probability that the estimate fall in the ROPE is 68% and the HDI 95% crosses the 0 threshold and panel D, where the probability of benefit is 100% and the 95%HDI and ROPE do not overlap.

The ROPE measures how much the posterior probability distribution falls between a specific interval of equivalent effect. By assessing how much of the 95% highest density interval (HDI) falls between the ROPE we can quantify the probability of the studied intervention having a benefit or harm. ^26^ We define the ROPE as the interval between 0.9 < OR > 1.1. In addition, we define a threshold for severe harm at OR = 1.25. We will draw samples from the posterior distribution after fitting the models with each of the previously defined priors and determine how much of the mass probability lies in the ROPE interval or exceed the threshold for severe harm and determine the expected predicted posterior probabilities of treatment effect using the *emmeans* package. Furthermore, we compare the ROPE interval with the 95% HDI as previously recommended to see if the HDI probability mass falls outside the ROPE. ^26^ To illustrate this principle, we simulate data for a binary outcome and a binary dependent variable with four different effect estimates and report the posterior distribution with 95%HDIs and ROPE along with probability masses after fitting logistic models with skeptic priors as previously stated and following a previously published approach ^7^ ( Figure 4).

### Subgroup analysis

To determine if the relationship between treatment and the primary outcome differs between predetermined, clinically significant subgroups, we will fit the varying effect logistic regression model by adding an interaction term between treatment and subgroup and report the conditional effect of the interaction.

We will assess the following subgroups:

• Type of surgery, i.e., laparoscopic vs. open surgery;
• Risk for PPCs, i.e., Assess Respiratory Risk in Surgical Patients in Catalonia (ARISCAT) score < 45 vs. ≥ 45;
• Body mass index (BMI), used as a continuous variable;
• Type of PEEP selection, i.e., fixed vs. titrated; and
• Use of a postoperative element as part of the tested intervention.

### Sensitivity analysis

We plan to perform the following sensitivity analyses:

• We will fit the same prespecified model but use only severe PPCs as the primary outcome, i.e. excluding mild respiratory failure.
• We will perform one analysis including only patients with lung collapse before the intervention; and
• We will fit the model by varying the heterogeneity prior τ to a to a half-Cauchy distribution with location 0 and scale 1. ^27^

## Ethics and dissemination

The study will be performed according to national and international guidelines. All data derive from clinical trials approved by a competent institutional review board in each participating center according to the applicable legislation. The study Steering Committee will publish the study findings. The writing committee will submit the main manuscript on behalf of the research group. All investigators listed in the original trials will be listed as collaborators in an Appendix in alphabetical order, according to the centre's name. All efforts will be made to link all collaborators to the final publication in indexed databases.

## Study status

We will carry out the analysis on the already locked database after publishing the protocol.

## Discussion

We here describe the protocol and statistical analysis plan for a Bayesian reanalysis of an individual patient data meta–analysis which aim is to compare the effect of intraoperative high PEEP after RM vs. low PEEP without RM on the incidence of postoperative pulmonary complications in patients undergoing surgery with general anesthesia and low tidal volume mechanical ventilation. The optimization of mechanical ventilation during surgery is important since it can potentially improve clinically relevant post-operative outcomes with beneficial effects on patients, families and healthcare systems.

The present proposed analysis has several strengths. Firstly, we can use the merged dataset of three well–performed multicenter RCTs, that tested the effect of fairly comparable intraoperative ventilation strategies concerning a similar endpoint. Secondly, the Bayesian framework will provide probabilities of harm or benefit associated with the studied intervention, adding further insights to the frequentist interpretations used in the previous analyses of these three RCTs. ^15^ We scrupulously followed the published recommendations, especially concerning prior selection. ^7^ Thirdly, we prespecify subgroups analysis to assess the intervention effect in particularly interesting subpopulations such as patients who underwent laparoscopic surgery, and sensitivity analyses to test the robustness of our methodology, which is crucial in a Bayesian framework.

The current standard statistical paradigm to analyze RCTs and perform meta–analyses is based on null–hypothesis testing and P–values and is referred to as frequentist approach. P–values indicate how incompatible a data set is with a specified statistical model, but their correct interpretation can be counterintuitive and at times even problematic. For instance, the typical P < 0.05 is defined as the probability that another study would yield a result equal to or more extreme than the one observed, assuming that the null hypothesis is true. This definition can hamper the correct interpretation of the results of different studies, particularly if P > 0.05. When a test returns a P > 0.05, a study is often interpreted as negative, meaning that the intervention had no effect on the outcome of interest, while the rigorous interpretation should be that the available data were insufficient to reject the null hypothesis. ^28^^,^^29^

The results from the REPEAT analysis fall precisely in this category. The effect of a high PEEP after RM maneuvers compared to low PEEP without RM on post-operative pulmonary complications was not statistically significant, with a P value of 0.06, thus yielding an indeterminate result. We plan to leverage the advantages of a Bayesian approach to expand and escape the all-or-nothing simplistic interpretation of the study derived from the null hypothesis testing. Further, a probabilistic framework to attach probabilities to specific estimates has been added to the analysis, thus providing a much more interpretable and intuitive explanation of the results.

Bayesian analysis in this context has proven to help gain additional insights and scope and has been increasingly used in recent years. For instance, Bayesian analysis applied to RCTs with indeterminate frequentist in intensive care setting interventions focused on improving mortality ^30^^,^^31^ found that the posterior probability of mortality benefit, i.e., relative risk (RR) < 1 or OR <1, ranged between 88% and 99% according to a range of prespecified priors. ^8^^,^^9^ Other reports used the same approach ^7^ to investigate further an opposite, i.e. probability of harm, indeterminate result in same clincal setting RCT, ^32^ found that the probability of harm of the intervention was > 90%. Bayesian analysis has also been used to elucidate the effect of interventions in specific subgroups, ^10^ to evaluate the effect of selection bias ^33^ and in meta-analysis. ^34^

This current analysis has several limitations which need to be addressed. First, some differences between studies concerning how PEEP and RM were used and titrated cannot be unraveled, although we will use a previously harmonized individual patient database. Secondly, we analyze the effect of a broad category, i.e., high PEEP and RM vs. low PEEP and no RM. Defining the optimal PEEP and RM strategy is beyond the scope of the current analysis and must be elucidated in further investigations. Thirdly, the original RCTs did not exclude patients without lung collapse; therefore, a selection bias towards less benefit of the intervention cannot be excluded. Fourthly, although previously published recommendations ^7^ were rigorously followed, there is no unequivocal way to choose a universally correct prior probability distribution. Moreover, although we included all data available from three of the largest RCTs assessing the effect of open lung strategy in the perioperative period, a certain degree of precision bias cannot be excluded should data from other studies be incorporated, although a change in the overall conclusions is unlikely.

In conclusion, we will use a Bayesian methodology to better interpret data from three large RCTs investigating the potential beneficial role of high PEEP after RM compared to low PEEP without RM during intraoperative low tidal mechanical ventilation to prevent post-operative pulmonary complications and improve clinically relevant outcome measures. Bayesian analysis can be a helpful tool to augment the interpretation of anesthesiology and critical care trials.

## Data availability

### Underlying data

The de-identified database derived from the original studies is stored at Hospital Israelita Einstein, Sao Paulo, Brazil. Data can be requested at the center IRB at: https://www.einstein.br/en/research/clinical-research-center/contact-us. Email address to contact: ary.neto2@einstein.br
