# Supplementary material for: Effect of intraoperative PEEP with recruitment maneuvers on the occurrence of postoperative pulmonary complications during general anesthesia––protocol for Bayesian analysis of three randomized clinical trials of intraoperative ventilation
Source: F1000Res. 2023 Jun 23;11:1090. Originally published 2022 Sep 22. [Version 2] doi: 10.12688/f1000research.125861.2 (PMC10207960; doi:10.12688/f1000research.125861.2)
Supplement: Peer review history

# Peer review: Reviewer response for version 2

**DOI:** 10.5256/f1000research.151851.r181196

**Published:** 2023-06-28

**Authors:** Lukas Gasteiger

**Affiliations:** 1 Department of Anesthesiology and Intensive Care, Universitat Innsbruck, Innsbruck, Tyrol, Austria

I thank the Authors for their response and recommend to accept the manuscript in its present form.

 Best regards

 Lukas Gasteiger

Is the study design appropriate for the research question?

Yes

Is the rationale for, and objectives of, the study clearly described?

Yes

Are sufficient details of the methods provided to allow replication by others?

Yes

Are the datasets clearly presented in a useable and accessible format?

Yes

Reviewer Expertise:

Ventilation, Right Ventricular Function, Circulation

I confirm that I have read this submission and believe that I have an appropriate level of expertise to confirm that it is of an acceptable scientific standard.

# Peer review: Reviewer response for version 1

**DOI:** 10.5256/f1000research.138209.r173629

**Published:** 2023-06-05

**Authors:** Ottokar Stundner

**Affiliations:** 1 Department of Anesthesia and Critical Care Medicine, Universitat Innsbruck, Innsbruck, Tyrol, Austria

The authors present a comprehensive protocol for a reanalysis of three RCTs using Bayesian meta-analysis techniques. While the three individual studies had slightly different scopes, the common primary outcome of all is the incidence of postoperative pulmonary complications (PPCs), and the exposure is a different ventilation strategy (“long protective” vs “non-lung protective”; high PEEP + recruitment maneuvers vs. low PEEP and no RMs).

 The authors argue that a recent meta-analysis using classical frequentist methodology (mixed effects logistic regression) yielded “no difference” between the exposure levels (n=1,913 “lung protective” vs. 1,924 “non-lung protective”, raw complication incidence 29.4% vs 32.2% (OR 0.87 (0.75-1.01)), but some differences in secondary endpoints (more frequent desaturations in low PEEP, more intraoperative hypotension in high PEEP, …). The point that Bayesian methodology, providing a more tangible and interpretable effect size for the interventions’ efficacy, would be a suitable analytic approach here, is well taken and there are prominent examples in the literature where a Bayesian re-analysis served to further the understanding of (particularly composite) datasets.

 Thank you for the opportunity to review your work. The protocol is very well written and the research question is valid, relevant, and succinctly presented.

 My two primary concerns lie (1) in the definition of PCCs, and (2) in the explanation text on how the effect estimate priors will be weighted.

 The PCC definition arguably differs between the three studies. E.g., the pulmonary infection category which is looser in PROVHILO than in the other studies, or bronchospasm, which is looser in PROBESE. The authors duly note the existence of these differences in their limitations section. As they argue, using a wide outcome definition may be sensible, given a relatively broad exposure bandwidth and heterogeneity in practical application of the treatment arms. The reviewer wonders if the authors deliberated adding different complication stages in the analysis or expanding their sensitivity analysis in this regard (they were already planning on excluding “minor” complications for sensitivity analysis). On the other hand, subgrouping may limit statistical power further and lead to model convergence issues.

 As for the prior definition; This important topic is explained at length and is planned according to a comprehensive protocol (Zampieri et al, 2021) and technical description (Röver et al, 2021). The reviewer appreciates the nuanced approach. However, I do believe that some more information / explanation could be conveyed regarding the choice of relative prior weighting.

 Specific comments:

- Please add a reference to the published results of the frequentist meta-analysis (Campos et al, BJA 2022 ^1^); only the study protocol is referenced.
- The authors will use a half-normal distribution for the heterogeneity prior. Could they briefly explain the rationale to perform a sensitivity analysis with I squared distribution (instead of i.e. Half-Cauchy).
- “Previous studies suggested a benefit of PEEP and RM, but many studies were neutral or indeterminate when using a frequentist approach. Therefore, we will consider a moderate belief strength for both the optimistic and neutral prior and a weak pessimistic prior. Following previous recommendations,7 we will define priors as follows….”
- What exactly does the word “therefore” signify here? It seems sensible to align the means ESs to the preexisting data, the weighting choice for the optimistic vs pessimistic priors could be explained some more (especially with regard to possible pessimistic assumptions, i.e., higher complication incidence). I suggest the authors elaborate a bit more here.

Is the study design appropriate for the research question?

Yes

Is the rationale for, and objectives of, the study clearly described?

Yes

Are sufficient details of the methods provided to allow replication by others?

Yes

Are the datasets clearly presented in a useable and accessible format?

Yes

Reviewer Expertise:

Comparative Effectiveness Research, Public Health, Regional Anesthesia

I confirm that I have read this submission and believe that I have an appropriate level of expertise to confirm that it is of an acceptable scientific standard.

## References

[1] : Intraoperative positive end-expiratory pressure and postoperative pulmonary complications: a patient-level meta-analysis of three randomised clinical trials. *Br J Anaesth* .2022;128(6) : 10.1016/j.bja.2022.02.039 1040-1051 10.1016/j.bja.2022.02.039 35431038

# Response

**Published:** 2023-06-20

**Authors:** Guido Mazzinari

**Affiliations:** University Hospital La Fe, Valencia, Spain

My two primary concerns lie (1) in the definition of PCCs, and (2) in the explanation text on how the effect estimate priors will be weighted.

 1. The PCC definition arguably differs between the three studies. E.g., the pulmonary infection category which is looser in PROVHILO than in the other studies, or bronchospasm, which is looser in PROBESE. The authors duly note the existence of these differences in their limitations section. As they argue, using a wide outcome definition may be sensible, given a relatively broad exposure bandwidth and heterogeneity in practical application of the treatment arms. The reviewer wonders if the authors deliberated adding different complication stages in the analysis or expanding their sensitivity analysis in this regard (they were already planning on excluding “minor” complications for sensitivity analysis). On the other hand, subgrouping may limit statistical power further and lead to model convergence issues.

**Thank you for the interesting remark. Indeed, we used a database where the primary outcome definition where previously harmonised to join the data from the different RCTs. New aspiration pneumonitis, pulmonary infiltrates and cardiogenic pulmonary oedema were not included in the definition of PPC, because they were not recorded in the iPROVE trial. Also, moderate respiratory failure, as defined only in the PROBESE trial was merged in the definition of severe respiratory failure to achieve concordance with the other trials. Still, there is some slight mismatch in some of the definitions, as the reviewer points out, and we already acknowledge in the limitations section of the protocol and in the previous studies. We planned indeed a sensitivity analysis excluding mild respiratory failure from the outcome to test the robustness of our findings. We purposedly tried to design a simple protocol and not overburden with too many secondary analysis because we feel that this would drive the focus away from the main question and could possibly complicate the model estimation as the reviewer correctly pointed out.**

 2. As for the prior definition, This important topic is explained at length and is planned according to a comprehensive protocol (Zampieri et al, 2021) and technical description (Röver et al, 2021). The reviewer appreciates the nuanced approach. However, I do believe that some more information / explanation could be conveyed regarding the choice of relative prior weighting.

**We agree that this is a key concept. We, therefore, reworked the paragraph on the prior to be clearer and more explicit. The updated version of the manuscript now reads as follows:**

*“For defining priors, we will follow previously published recommendations on Bayesian reanalysis ^7^ and recommendations from studies focusing on Bayesian modelling and meta–analysis. ^22,23^ The guidelines recommend to consider the full range of possible beliefs. Thus, we will use one skeptical, one pessimistic, and one optimistic prior for the effect of PEEP and RM compared to low PEEP, i.e., a PEEP of 5 cmH2O or less, without RM on PPCs. In other words, we define three distributions to decide where to assign most of the probability mass. The pessimistic and optimistic prior distributions will be centred, on the averaged estimate from the original, whereas the skeptical prior will be centred around the absence of effect, i.e. 0. *The variance of the prior distributions is defined according to the clinical belief that we cannot rule out a benefit for the intervention, although we can likely rule out a large effect and we cannot exclude a probability of harm and we set the probability masses accordingly. Therefore, we will consider a moderate belief strength for both the optimistic and neutral prior and a weak pessimistic prior. Following previous recommendations, ^7^ we will define priors as follows:

- The neutral skeptical prior of moderate strength is normally distributed and centered at the absence of effect [OR = 1; log (OR) = 0] with an SD of 0.355, such that 0.95 of the probability falls in the range of 0.5–2. Therefore, our skeptical prior will follow a normal distribution with a mean of 0 and an SD of 0.355 [N(0, 0.355)] (Figure 1);
- The pessimistic and optimistic priors are informed by the averaged effect size estimate from the three original studies (PROVHILO:OR= 0.63; iPROVE:OR= 0.45; PROBESE:OR= 0.44; Mean:OR= 0.51). The standard deviation (SD) of the optimistic prior will be defined to retain a 0.15 probability of harm [Pr (OR > 1)], and the pessimistic prior is chosen to retain a 0.30 probability of benefit [Pr (OR < 1)] (Figure 1)”

Specific comments:

 3. Please add a reference to the published results of the frequentist meta-analysis (Campos et al, BJA 2022 1); only the study protocol is referenced.

**The mentioned study is already referenced in the manuscript; it is reference #15. If the reviewer is referencing another study, please let us know, and we will add it to the manuscript.**

 4. The authors will use a half-normal distribution for the heterogeneity prior. Could they briefly explain the rationale to perform a sensitivity analysis with I squared distribution (instead of i.e. Half-Cauchy).

**We agree. The I ^2^ distribution is used mainly for modelling heterogeneity in conventional Bayesian metanalysis models with aggregate data, as explained in length in Rover et al. Res. Synth. Methods. 2021 Jul 1;12(4): 448–474. Following the reviewer, we changed the distribution of the heterogeneity prior for the sensitivity analysis to a half-Cauchy prior. We changed the manuscript accordingly, which now reads:**

 “We will fit the model by varying the heterogeneity prior τ to a half-Cauchy distribution with location 0 and scale 1."

 5.“Previous studies suggested a benefit of PEEP and RM, but many studies were neutral or indeterminate when using a frequentist approach. Therefore, we will consider a moderate belief strength for both the optimistic and neutral prior and a weak pessimistic prior. Following previous recommendations,7 we will define priors as follows….”. What exactly does the word “therefore” signify here? It seems sensible to align the means ESs to the preexisting data, the weighting choice for the optimistic vs pessimistic priors could be explained some more (especially with regard to possible pessimistic assumptions, i.e., higher complication incidence). I suggest the authors elaborate a bit more here.

**Please see our reply to comment #2. We hopefully made the logic behind the priors definition clearer.**

# Peer review: Reviewer response for version 1

**DOI:** 10.5256/f1000research.138209.r173633

**Published:** 2023-05-22

**Authors:** Lukas Gasteiger

**Affiliations:** 1 Department of Anesthesiology and Intensive Care, Universitat Innsbruck, Innsbruck, Tyrol, Austria

This is a very well designed protocol for a Bayesian analysis of three recent RCT's to better understand the effect of intraoperative PEEP on PPCs. By now no RCT was able to clearly show a benefit of a higher PEEP and RM compared to lower PEEP levels and no RM. Therefore the question, if such an intervention is beneficial to our patients, is of high clinical relevance. 

 The chosen model to use a Bayesian protocol seems appropriate and adds the advantage that no new trial and inclusion of patients is needed.

 Furthermore to assess the effect of this intervention is of central interest as it is known, that a higher lever of PEEP may also interact with Right Ventricular Function and therefore may lead to circulatory side-effects such as hypotension. Also, an increased need for vasopressor therapy may be associated. 

- Is it planned to also assess for circulatory side-effects as secondary outcome?

Is the study design appropriate for the research question?

Yes

Is the rationale for, and objectives of, the study clearly described?

Yes

Are sufficient details of the methods provided to allow replication by others?

Yes

Are the datasets clearly presented in a useable and accessible format?

Yes

Reviewer Expertise:

Ventilation, Right Ventricular Function, Circulation

I confirm that I have read this submission and believe that I have an appropriate level of expertise to confirm that it is of an acceptable scientific standard.

# Response

**Published:** 2023-05-23

**Authors:** Guido Mazzinari

**Affiliations:** University Hospital La Fe, Valencia, Spain

Thank you very much for your time and effort in reviewing our protocol.

 Regarding your question:

**- Is it planned to also assess for circulatory side-effects as secondary outcome?**

 We chose to limit this reanalysis to the primary outcome, i.e. occurrence of postoperative pulmonary complications, to keep our aim simple and focused. Moreover, we feel that the prior distribution selection was easier since we followed a previously published approach to this type of outcome. As for hypotension episodes of hemodynamic related side-effects, the longitudinal nature of the data would require a careful planning of the priors, and we are not aware of any already accepted approach in this field. It is certainly worth looking into it in the future.

# Peer review: Reviewer response for version 1

**DOI:** 10.5256/f1000research.138209.r173632

**Published:** 2023-05-22

**Authors:** Andrea Cortegiani

**Affiliations:** 1 Department of Surgical, Oncological and Oral Science, Universita degli Studi di Palermo, Palermo, Sicily, Italy

The authors submitted the protocol for a Bayesian analysis of a dataset composed by data from three major RCTc of intraoperative ventilation evaluating high PEEP + recruitment maneuvers vs low PEEP and no recruitment maneuvers. The dataset (REPEAT) has been already used for frequentist analysis and is of high internal and external validity. The authors are expert in the field and are able to provide important insights with this analysis. The methods for this Bayesian analysis, especially the selected prior distributions are reasonable and adequate.

 I fully support the indexing of this protocol and look forward to seeing the results of the analysis.

Is the study design appropriate for the research question?

Yes

Is the rationale for, and objectives of, the study clearly described?

Yes

Are sufficient details of the methods provided to allow replication by others?

Yes

Are the datasets clearly presented in a useable and accessible format?

Yes

Reviewer Expertise:

Anaesthesiology and Critical Care Medicine

I confirm that I have read this submission and believe that I have an appropriate level of expertise to confirm that it is of an acceptable scientific standard.

# Response

**Published:** 2023-05-23

**Authors:** Guido Mazzinari

**Affiliations:** University Hospital La Fe, Valencia, Spain

Thank you very much for your time and effort to review our protocol.
